# Gustave Roussy Immune Score (GRIm-Score) is a prognostic marker in patients with resectable esophageal squamous cell carcinoma

**DOI:** 10.7150/jca.37898

**Published:** 2020-01-01

**Authors:** Ji-Feng Feng, Liang Wang, Xun Yang, Sheng Chen

**Affiliations:** 1 Department of Thoracic Oncological Surgery, Institute of Cancer Research and Basic Medical Sciences of Chinese Academy of Sciences, Cancer Hospital of University of Chinese Academy of Sciences, Zhejiang Cancer Hospital, No.38 Guangji Road, Hangzhou 310022, China.; 2 Key Laboratory Diagnosis and Treatment Technology on Thoracic Oncology, No.38 Guangji Road, Zhejiang province, Hangzhou 310022, China.

**Keywords:** esophageal squamous cell carcinoma, neutrophil to lymphocyte ratio, albumin, lactate dehydrogenase, prognosis

## Abstract

**Background**: The Gustave Roussy Immune Score (GRIm-Score) was initially reported to select patients for immunotherapy. Therefore, the purpose of the current retrospective study was to determine whether GRIm-Score, a novel nutritional and inflammatory-based prognostic score, is a useful prognostic marker in patients with esophageal squamous cell carcinoma (ESCC) undergoing surgical resection.

**Methods**: A retrospective single institutional study including 372 ESCC patients undergoing surgical resection was performed. The GRIm-Score was simply calculated by lactate dehydrogenase (LDH), neutrophil lymphocyte ratio (NLR) and albumin (ALB). The cancer-specific survival (CSS) was analyzed for the current study with Cox regression analyses with forward stepwise and Kaplan-Meier methods.

**Results**: There were 284 (76.3%) men and 88 (23.7%) women with the mean age of 59.3 ± 8.0 years (range: 36-80 years). Patient with a high GRIm-Score had poor CSS (10.3% vs. 35.0%, *P* < 0.001). The GRIm-Score, in multivariate analyses, instead of NLR, LDH or ALB, was an independent prognostic factor for CSS (*P* = 0.004).

**Conclusion**: The GRIm-Score was an independent prognostic marker in patients with ESCC undergoing surgical resection. Our study is also the first study to discuss the prognostic value of GRIm-Score in patients with resectable ESCC.

## Introduction

As one of the leading malignant tumors worldwide, esophageal cancer (EC) is common in China with 477,900 new cases and 375,000 deaths 1. Esophageal squamous cell carcinoma (ESCC) is the main pathological type of EC in China 2. Although there are many therapies for patients with ESCC, the prognosis for patients with ESCC remains poor 3, 4. Surgical resection remains the first choice for patients with early-stage disease, while chemoradiotherapy is the mainstay treatment in patients with local advanced disease. Therefore, it is very necessary to find more useful and effective preoperative clinical variables for patients with ESCC.

The nutrition and inflammation play an important role in cancer prognosis 5. Some studies revealed that neutrophil to lymphocyte ratio (NLR) and C-reactive protein (CRP) were associated with prognosis in several types of cancers, including EC 6-9. As an important nutritional factor, albumin (ALB) reflected the nutritional status in a variety of cancers. Some studies published in recent years revealed that ALB was still a controversial prognostic factor in patients with EC 10, 11. It has been reported that lactate dehydrogenase (LDH) may act as an indicator for tumor burden and aggressiveness 12, 13. Patients with high level of serum LDH have worse prognosis, but remains controversial in ESCC 14-16.

Recently, the Gustave Roussy Immune Score (GRIm-Score) was firstly identified with the purpose of a better patient selection in clinical trials for immunotherapy 17. The results demonstrated that the GRIm-Score, based on NLR, LDH and ALB, is a better prognostic marker for patients enrolled in experimental trials. Moreover, the prognostic value of GRIm-Score has been confirmed in non-small cell lung cancer 18, 19. However, as far as we know, there are no studies regarding the prognostic value of GRIm-Score in patients with ESCC so far. The purpose of the current study was to evaluate the prognostic value of GRIm-Score in patients with resectable ESCC.

## Materials and Methods

### Patients

We retrospectively analyzed the clinical characteristics of 372 resectable patients with ESCC who undergoing curative esophagectomy between January 2006 and December 2010. This was a single institutional study. The inclusion criteria were as follow: (1) ESCC with stage I-III was confirmed by histopathology, (2) curative esophagectomy was performed without any neoadjuvant chemoradiotherapy, and (3) clinical characteristics and preoperative laboratory results, such as neutrophil, lymphocyte, LDH, ALB and CRP, were obtained before surgery within one week. The study was approved by the Ethics Committees, and written informed consent for the collection of specimen was obtained from each patient. The last follow up time was June 2014.

### Data collection

The main clinical characteristics requiring analysis were collected from our medical records. The levels of neutrophil, lymphocyte, LDH, ALB and CRP were obtained within one week before surgery. The 7th AJCC/UICC TNM staging system was used in the current study 20. The GRIm-Score is calculated by the following three variables: LDH (within normal range: 0 vs. > upper limit of normal (ULN) of each center, 240 U/L in our hospital: +1), ALB (≥ 35 g/L: 0 vs. < 35g/L: +1), and NLR (≤ 6: 0 vs. > 6: +1). Patients were divided into two groups: high group (score 2 or 3) and low group (score 0 or 1) 17.

### Statistical analyses

The Chi-squared test (for categorical variables), Student's t-test (for continuous variables with Gaussian distribution) and Mann-Whitney U test (for continuous variables without Gaussian distribution) were performed to compare the clinical characteristics grouped by GRIm-Score. The endpoint for the current study was cancer-specific survival (CSS). The Kaplan-Meier method was used to compare the CSS by using the log-rank test. Multivariate analyses with Cox regression analyses by forward stepwise regression were used to evaluate the independent prognostic factors. The areas under the curve (AUC) for NLR, LDH, ALB, CRP and GRIm-Score were compared and calculated with the receiver operating characteristic (ROC) curves. A nomogram model with R 3.6.0 software was used to predict the 1-, 3- and 5-year CSS probability by using the independent prognostic factors in multivariate analyses 21. All statistical analyses were performed with MedCalc 15.2 (MedCalc Software bvba, Ostend, Belgium) and SPSS 20.0 (SPSS Inc., Chicago, IL, USA).

## Results

### Patient Characteristics

**Figure 1:**
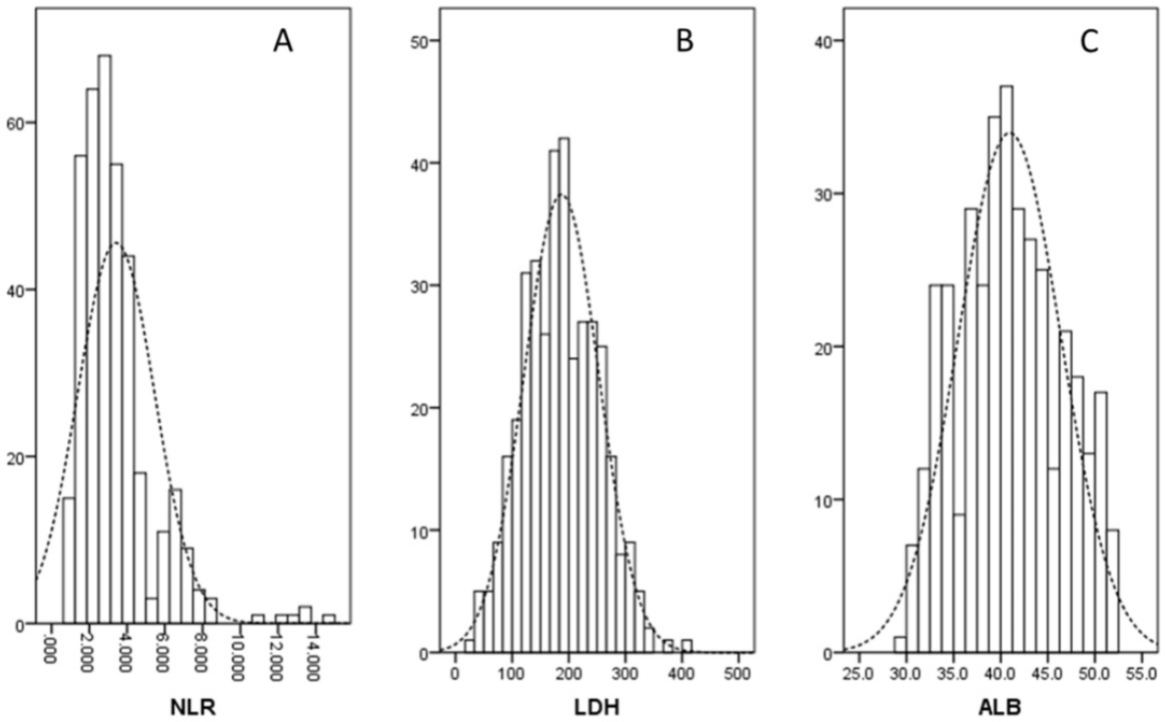
The histograms of the NLR (A), LDH (B) and ALB (C).

**Figure 2:**
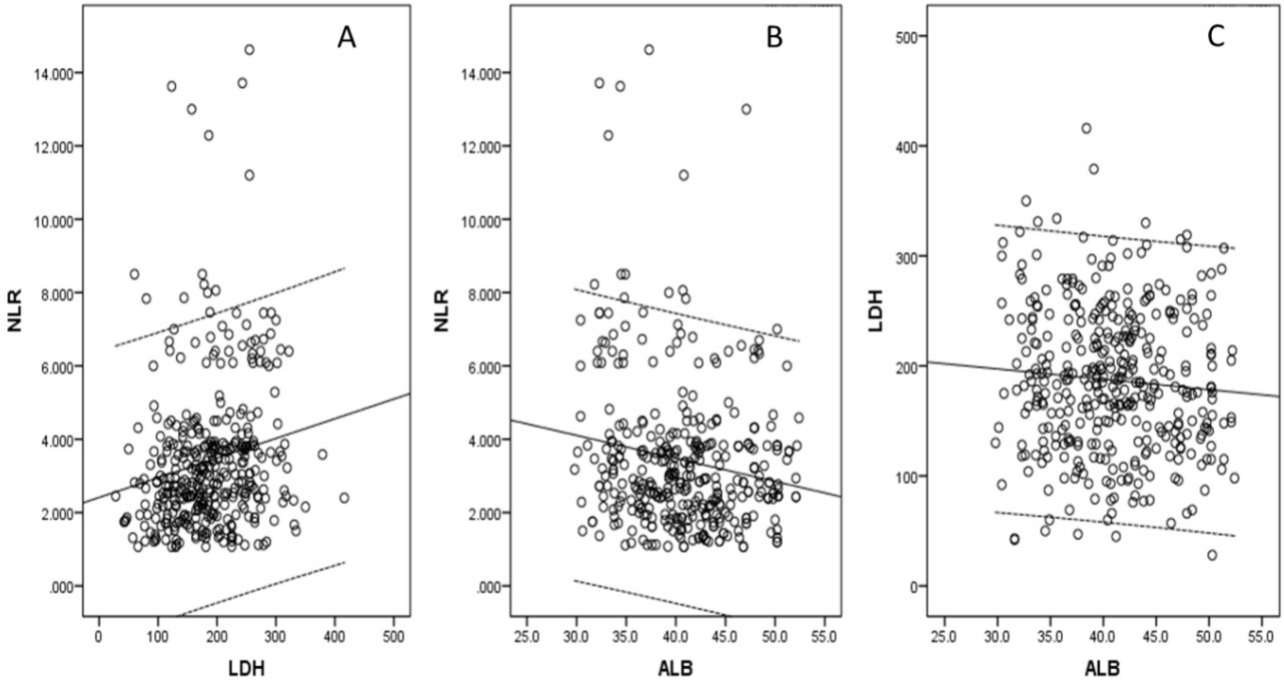
** Correlations for NLR, LDH and ALB.** Negative correlations between NLR and ALB (A). Positive correlations between NLR and LDH (B). No correlations were found between ALB and LDH (C).

There were 284 (76.3%) men and 88 (23.7%) women with the mean age of 59.3 ± 8.0 years (range: 36-80 years). According to clinical criteria, a total of 115 patients (30.9%) received postoperative adjuvant radiotherapy and/or chemotherapy. The histograms of the NLR, LDH and ALB were shown in Figure 1. There were negative correlations between NLR and ALB (r = -0.169, *P* = 0.001; Fig. 2A), positive correlations between NLR and LDH (r = 0.174, *P* = 0.001; Fig. 2B), but no correlations between ALB and LDH (r = -0.078, *P* = 0.135; Fig. 2C). The clinical characteristics regarding GRIm-Score were shown in Table 1.

### CSS analyses

**Figure 3:**
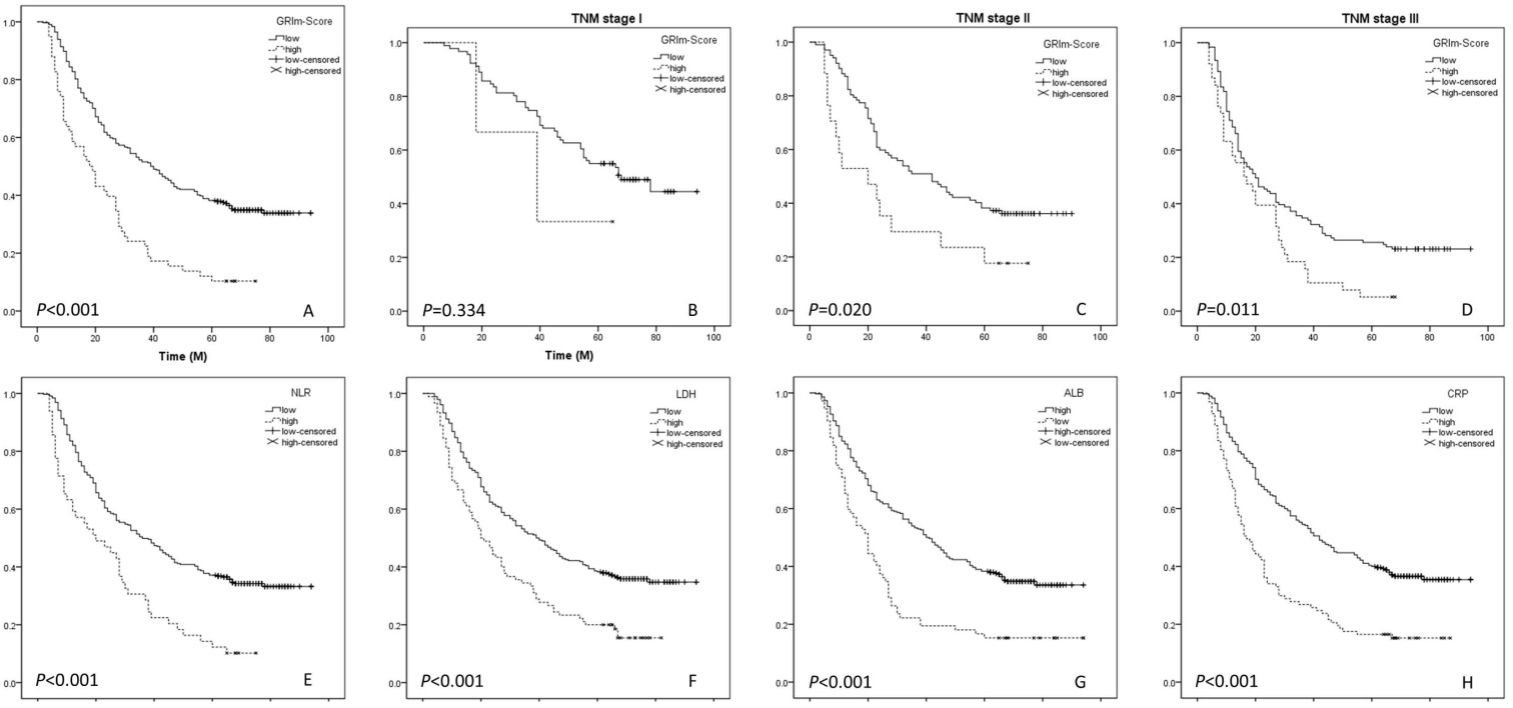
** CSS analyses.** Kaplan-Meier for CSS grouped by GRIm-Score (A). CSS analyses for GRIm-Score in subgroup analyses based on TNM stage (B-D). Kaplan-Meier for CSS grouped by NLR (E), LDH (F), ALB (G) and CRP (H).

At the last follow-up time, 256 (68.8%) of the 372 patients had died. Patients with a high GRIm-Score had poor 5-year CSS (10.3% vs. 35.0%, *P* < 0.001) (Fig. 3A). In subgroup analyses based on TNM stage (TNM I, TNM II and TNM III), we revealed that GRIm-Score was also significantly related to CSS in TNM II (*P* = 0.020) and TNM III (*P* = 0.011), but not in TNM I (*P* = 0.334) (Fig. 3B-D). The significantly differences for 5-year CSS were also found in NLR (34.4% vs. 10.2%, *P* < 0.001), ALB (35.0% vs. 15.3%, *P* < 0.001), LDH (35.8% vs. 16.7%, *P* < 0.001) and CRP (36.7% vs. 15.5%, *P* < 0.001) (Fig. 3E-H).

### Cox regression analyses

Univariate analyses indicated that several clinical indexes, such as vessel invasion, perineural invasion, tumor length, TNM stage, CRP, ALB, NLR, LDH and GRIm-Score, were significant predictors of CSS (Table 2). Multivariate analyses demonstrated that GRIm-Score (HR: 1.593, 95% CI: 1.156-2.197, *P* = 0.004), instead of NLR, LDH or ALB, was an independent prognostic factor (Table 3). Furthermore, CRP (HR: 1.760, 95% CI: 1.339-2.314, *P* < 0.001) and TNM stage (HR: 1.478, 95% CI: 1.027-2.129, *P* = 0.036 and HR: 2.364, 95% CI: 1.676-3.332, *P* < 0.001) were other significant prognostic factors (Table 3).

### ROC curve analyses

**Figure 4:**
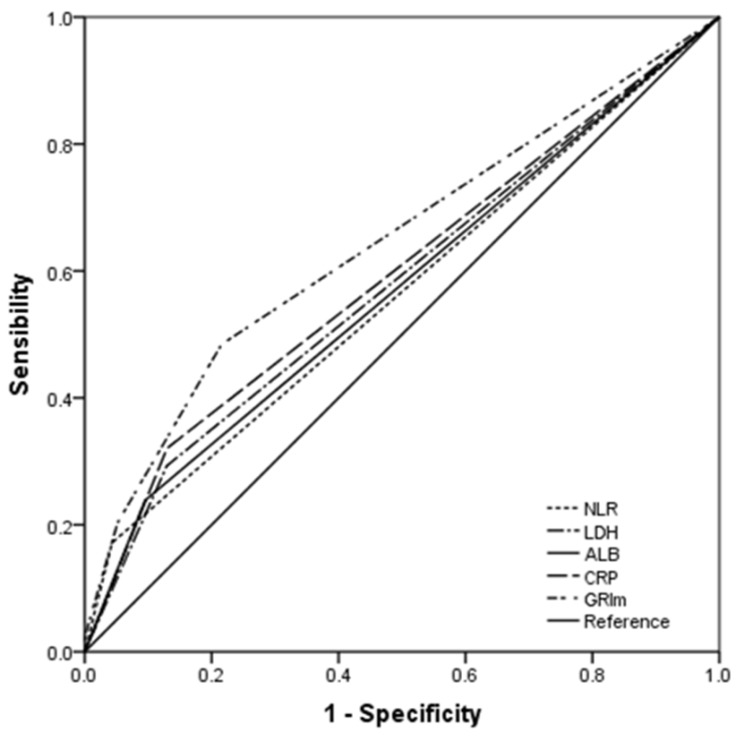
** ROC analyses.** The AUC area of the GRIm-Score (0.644) was higher than that of CRP (0.596), NLR (0.564), ALB (0.572) and LDH (0.582) for all the ESCC patients.

The AUC areas were 0.644, 0.596, 0.564, 0.572 and 0.582 for GRIm-Score (95% CI: 0.593-0.693), CRP (95% CI: 0.544-0.646), NLR (95% CI: 0.512-0.615), ALB (95% CI: 0.520-0.623) and LDH (95% CI: 0.530-0.632), respectively (Fig. 4). Comparison of AUC areas regarding the GRIm-Score, CRP, NLR, ALB and LDH in ESCC were shown in Table 4.

### ROC curve analyses

The AUC areas were 0.644, 0.596, 0.564, 0.572 and 0.582 for GRIm-Score (95% CI: 0.593-0.693), CRP (95% CI: 0.544-0.646), NLR (95% CI: 0.512-0.615), ALB (95% CI: 0.520-0.623) and LDH (95% CI: 0.530-0.632), respectively (Fig. 4). Comparison of AUC areas regarding the GRIm-Score, CRP, NLR, ALB and LDH in ESCC were shown in Table 4.

### Nomogram analyses

**Figure 5:**
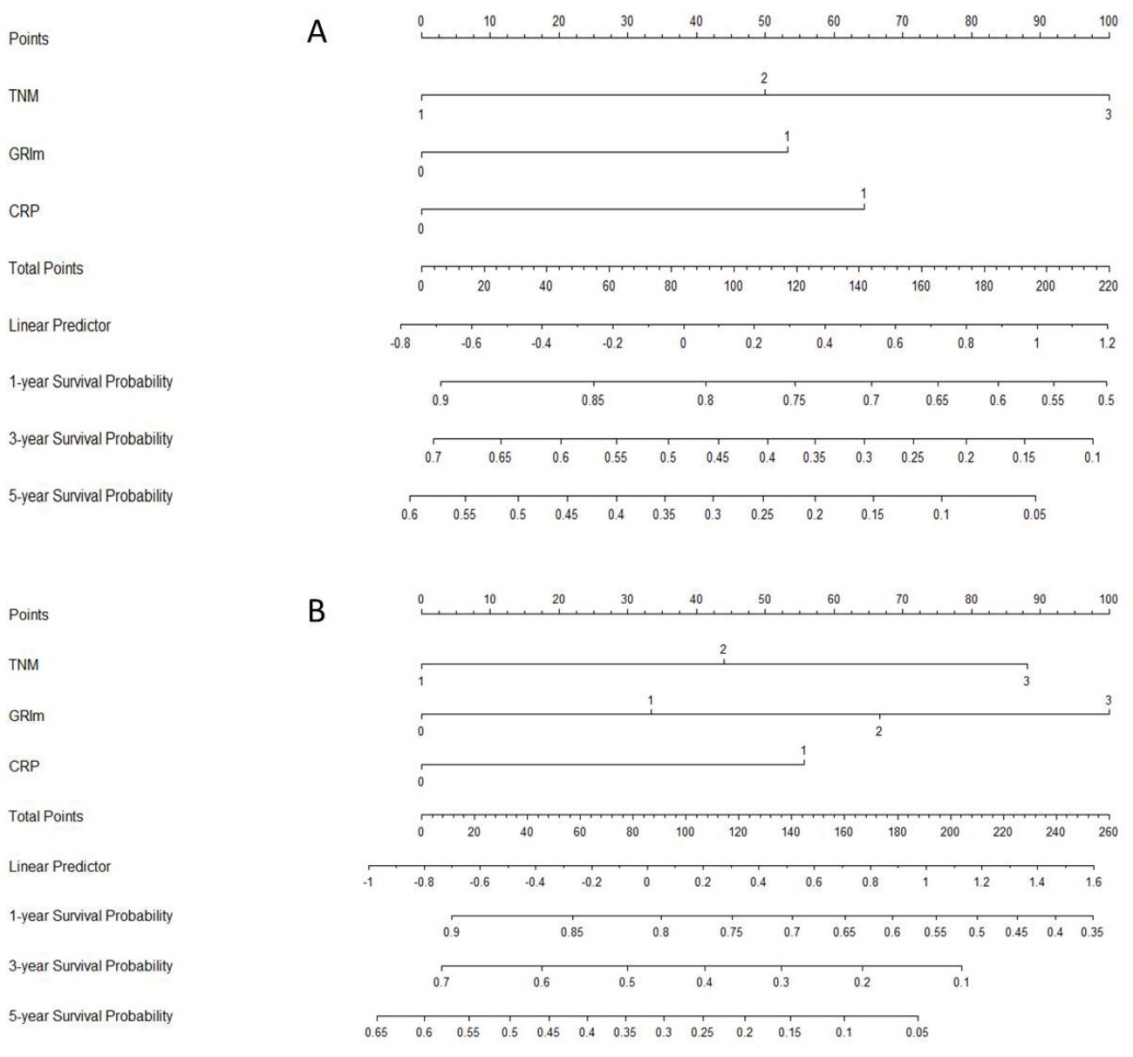
** Nomogram analyses.** Two nomogram models were conducted for different GRIm-Score [GRIm-Score 0, 1 (A) and GRIm-Score 0, 1, 2, 3 (B)] to predict the 1-, 3- and 5-year CSS probability.

Moreover, a nomogram model, including the significant prognostic factors (GRIm-Score, TNM and CRP), was conducted to predict the 1-, 3- and 5-year CSS probability for patients with ESCC (Fig. 5). Two nomogram models were conducted for different GRIm-Score (GRIm-Score 0, 1 and GRIm-Score 0, 1, 2 and 3).

## Discussion

This study, as far as we know, is the first study in patients with ESCC to indicate the prognostic value of GRIm-Score. The current study revealed several important findings: (1) GRIm-Score was a strong prognostic marker for CSS; (2) GRIm-Score, instead of NLR, ALB or LDH, was a useful independent prognostic factor. Therefore, we concluded that the GRIm-score was not only a selective biomarkers for patients enrolled in clinical trials for immunotherapy, but also a useful prognostic biomarkers for ESCC patients undergoing surgical resection.

The GRIm-Score was firstly identified with the purpose of a better patient selection in clinical trials for immunotherapy 17. The results demonstrated that the GRIm-Score, based on NLR, LDH and ALB, is a better prognostic marker for patients enrolled in experimental trials. Moreover, the prognostic value of GRIm-Score has been confirmed in non-small cell lung cancer in recent years 18, 19. However, as far as we know, there are no studies regarding the prognostic value of GRIm-Score in ESCC patients so far. In our study, patients with a high GRIm-Score had worse 5-year CSS (10.3% vs. 35.0%, *P* < 0.001). Multivariate analyses demonstrated that GRIm-Score (HR: 1.593, 95% CI: 1.156-2.197, *P* = 0.004), instead of NLR, LDH or ALB, was an independent prognostic factor.

The NLR, ALB and LDH are routine laboratory indicators in daily clinical practice. Recent studies reported that inflammation is associated with poor prognosis in cancers, with NLR as a sensitive inflammatory biomarker in several types of cancers, including EC 6-9. The levels of ALB, as an important nutritional factor, reflected the nutritional status in a variety of cancers. Some studies published in recent years revealed that ALB was still a controversial prognostic factor in patients with EC 10, 11. Patients with high level of serum LDH have worse prognosis, but remains controversial in ESCC 14-16. A retrospective study including 906 patients with ESCC concluded that LDH was associated with overall survival (OS) with the optimal cutoff point of 361.5 U/L 14. However, a total of 212 patients with ESCC undergoing chemoradiotherapy revealed that LDH (cutoff point: 170 U/L) was not associated with OS or progression-free survival (PFS) 15. The results were consistent with another study including 447 patients with ESCC which revealed that LDH (cutoff point: 154.4 U/L) was not a prognostic factor regarding OS 16.

It should be noted that NLR, ALB and LDH, routine laboratory indicators in daily clinical practice, may be influenced by various conditions. In the present study, although NLR, ALB and LDH were found to be predictive factors for CSS in univariate analyses, multivariate analyses did not show these biomarkers to be independent prognostic factors. Therefore, GRIm-Score is a combined indicator which can reflect a mixed prognostic value.

It should be also noted that our results have potential clinical significance in the treatment of patients with ESCC. Patients in a high level of GRIm-Score with early-stage ESCC may require more frequent follow-up, while those with local advanced ESCC may need more adjuvant therapy. However, the results of our study should be validated in more large-sample prospective trials in future.

Some potential limitations of the current study should be mentioned. Firstly, the potential selection bias should be acknowledged due to the current study was a retrospective research with single center. Secondly, patients with preoperative treatment in the current study were excluded, which might have influenced the results for patients with ESCC who underwent surgical resection. As everyone knows, preoperative neoadjuvant therapy will have side effects on laboratory indicators. However, preoperative neoadjuvant therapy (chemotherapy and/or radiotherapy) followed by surgery improves survival in several randomized control trials for locally advanced disease of EC, but not for early-stage EC 22, 23. Thirdly, due to lack of prospective study, the results of our study should be validated in more large-sample clinical trials in future. In the current study, a nomogram model, including the significant prognostic factors (GRIm-Score, TNM and CRP), was conducted to predict the 1-, 3- and 5-year CSS probability for patients with ESCC. However, we must acknowledge that our prognostic nomogram model was only developed, but not validated in this study. Therefore, the results of our study should be validated in future.

In summary, the GRIm-Score, a novel nutritional and inflammatory-based prognostic score, was an independent prognostic factor in patients with ESCC undergoing surgical resection. The current study, as far as we know, is the first study regarding the prognostic value of GRIm-Score in ESCC patients undergoing esophagectomy.

## Figures and Tables

**Table 1:** Clinical characteristics based on GRIm-Score in patients with ESCC.

	Total (n = 372)	Low (n = 314)	High (n = 58)	P-value
Age (years)				
Mean ± SD	59.3 ± 8.0	59.5 ± 8.0	58.3 ± 7.7	0.322^#^
≤ 60 / > 60 (n)	210 / 162	176 / 138	34 / 24	0.717
Gender (n)				
Male / Female	284 / 88	238 / 76	46 / 12	0.563
Tumor length (cm)				
Median (IQR)	4.0 (3.0-5.3)	4.0 (3.0-5.0)	5.0 (4.0-6.3)	< 0.001*
≤ 3.0 / > 3.0 (n)	106 / 266	97 / 217	9 / 49	0.017
Tumor location (n)				
Upper/ Middle/ Lower	25 / 172 / 175	22 / 142 / 150	3 / 30 / 25	0.632
Vessel invasion (n)				
Negative / Positive	312 / 60	264 / 50	48 / 10	0.802
Perineural invasion (n)				
Negative / Positive	293 / 79	250 / 64	43 / 15	0.348
Smoking (n)				
No / Yes	203 / 169	169 / 145	34 / 24	0.500
Drinking (n)				
No / Yes	228 / 144	193 / 121	35 / 23	0.872
Differentiation (n)				
Well/ Moderate/ Poor	52 / 246 / 74	47 / 208 / 59	5 / 38 / 15	0.266
TNM stage (n)				
I / II / III	94 / 119 / 159	91 / 102 / 121	3 / 17 / 38	< 0.001
NLR				
Median (IQR)	2.93 (2.16-3.87)	2.74 (1.95-3.67)	6.32 (4.09-7.09)	< 0.001^*^
≤ 6 / > 6 (n)	323 / 49	305 / 9	18 / 40	< 0.001
LDH (U/L)				
Mean ± SD	187.0 ± 66.1	177.2 ± 62.7	240.1 ± 58.7	< 0.001^#^
≤ 240 / > 240 (n)	282 / 90	264 / 50	18 / 40	< 0.001
ALB (g/L)				
Mean ± SD	40.9 ± 5.5	41.7 ± 5.1	36.7 ± 5.6	< 0.001^#^
> 35 / ≤ 35 (n)	300 / 72	284 / 30	16 / 42	< 0.001
CRP (mg/L)				
Median (IQR)	4.55 (1.96-10.26)	4.46 1.72-8.39)	7.52 (3.43-15.58)	< 0.001^*^
≤ 10 / > 10 (n)	275 / 97	242 / 72	33 / 25	< 0.001
Adjuvant therapy				
No / Yes	257 / 115	220 / 94	37 / 21	0.342

ESCC: esophageal squamous cell carcinoma; GRIm-Score: Gustave Roussy Immune Score; CRP: C-reactive protein; ALB: albumin; LDH: lactate dehydrogenase; NLR: neutrophil to lymphocyte ratio; TNM: tumor node metastasis; SD: standard deviation; IQR: interquartile range. ^#^: t-test; ^*^: Mann-Whitney U test.

**Table 2:** Univariate analyses of CSS in ESCC patients.

	5-year CSS	Mediean (M)	P-value	HR (95% CI)	P-value
Age (years)			0.997		0.997
≤ 60	30.5%	32		1.000	
> 60	32.1%	32		1.000 (0.780-1.280)	
Gender			0.950		0.951
female	31.8%	28		1.000	
male	31.0%	32		1.009 (0.756-1.348)	
Tumor length (cm)			0.003		0.004
≤ 3.0	39.6%	48		1.000	
> 3.0	27.8%	27		1.522 (1.147-2.021)	
Tumor location			0.381		0.392
upper	44.0%	45		1.000	
middle	29.1%	32		1.471 (0.846-2.558)	0.172
lower	31.4%	32		1.402 (0.806-2.439)	0.231
Vessel invasion			0.015		0.017
negative	33.3%	36		1.000	
positive	20.0%	20		1.469 (1.073-2.013)	
Perineural invasion			0.006		0.007
negative	34.1%	39		1.000	
positive	20.3%	20		1.486 (1.117-1.977)	
Smoking			0.265		0.271
no	34.0%	35		1.000	
yes	27.8%	27		1.148 (0.898-1.467)	
Drinking			0.606		0.610
no	30.3%	36		1.000	
yes	32.6%	25		1.068 (0.830-1.375)	
Differentiation			0.142		0.150
well	38.5%	35		1.000	
moderate	30.9%	34		1.118 (0.766-1.631)	0.564
poor	27.0%	16		1.461 (0.943-2.263)	0.089
TNM stage			<0.001		<0.001
I	48.9%	67		1.000	
II	33.6%	34		1.697 (1.185-2.430)	0.004
III	18.9%	19		2.784 (1.995-3.886)	<0.001
CRP (mg/L)			<0.001		<0.001
≤ 10.0	36.7%	42		1.000	
> 10.0	15.5%	17		2.060 (1.581-2.685)	
ALB (g/L)			<0.001		<0.001
≥ 35.0	35.0%	40		1.000	
< 35.0	15.3%	20		1.979 (1.480-2.645)	
NLR			<0.001		<0.001
≤ 6.0	34.4%	36		1.000	
> 6.0	10.2%	20		2.029 (1.464-2.812)	
LDH (U/L)			<0.001		<0.001
≤ 240	35.8%	39		1.000	
> 240	16.7%	20		1.767 (1.348-2.315)	
GRIm-Score			<0.001		<0.001
low	35.0%	39		1.000	
high	10.3%	18		2.246 (1.652-3.054)	
Adjuvant therapy			0.129		0.133
No	32.3%	38		1.000	
Yes	28.7%	23		1.223 (0.940-1.591)	

ESCC: esophageal squamous cell carcinoma; CSS: cancer-specific survival; GRIm-Score: Gustave Roussy Immune Score; CRP: C-reactive protein; ALB: albumin; LDH: lactate dehydrogenase; TNM: tumor node metastasis; NLR: neutrophil to lymphocyte ratio; CI: confidence interval; HR: hazard ratio.

**Table 3:** Multivariate analyses of CSS in patients with ESCC.

	HR (95% CI)	P-value
TNM stage		<0.001
I	1.000	
II	1.478 (1.027-2.129)	0.036
III	2.364 (1.676-3.332)	<0.001
GRIm-Score		0.004
low	1.000	
high	1.593 (1.156-2.197)	
CRP (mg/L)		<0.001
≤ 10.0	1.000	
> 10.0	1.760 (1.339-2.314)	

ESCC: esophageal squamous cell carcinoma; CSS: cancer-specific survival; GRIm-Score: Gustave Roussy Immune Score; CRP: C-reactive protein; TNM: tumor node metastasis; CI: confidence interval; HR: hazard ratio.

**Table 4:** Comparison of AUC areas for the prognostic factors in ESCC.

	AUC	95% CI	P-value
GRIm-Score	0.644	0.593-0.693	Reference
CRP	0.596	0.544-0.646	0.0897
NLR	0.564	0.512-0.615	0.0001
ALB	0.572	0.520-0.623	0.0001
LDH	0.582	0.530-0.632	0.0004

ESCC: esophageal squamous cell carcinoma; GRIm-Score: Gustave Roussy Immune Score; CRP: C-reactive protein; ALB: albumin; LDH: lactate dehydrogenase; NLR: neutrophil to lymphocyte ratio; AUC: area under the curve.
